# Effects of Rhubarb and Santonin on the Urine

**Published:** 1879-01

**Authors:** 


Effects of Rhubarb and Santonin on the Urine.—Dr. J Munk (*Virchow's Archiv*) found that after the internal administration of both rhubarb and santonin the urine was of a greenish color, and that in both cases the addition of alkalies changes this to red. Notwithstanding this resemblance there are points of difference:

First. Alkaline carbonates produce the redish color almost instantly after rhubarb has been taken, while after santonin the change is exceedingly slow.

Second. This change of color of the rhubarb urine by alkalies is permanent; that after santonin passes away in twenty-four to forty-eight hours (if caustic soda be employed it may last longer).

Third. The rhubarb urine, colored red by alkalies, is discolored by digestion with steel filings; that after santonin is not.

Fourth. By adding to the rhubarb urine an excess of baryta or lime water, and filtering, the deposit retains the reddish color, and the filtrate remains clear; on the contrary, in santonin urine the pigment remains in solution, leaving us a reddish filtrate and an uncolored sediment.—*Druggist's Circular and Chemical Gazette.*

